# Free Vascularized Fibular Graft Transfer in the Reconstruction of Defects for Premalignant and Malignant Musculoskeletal Conditions of the Femur in a Tertiary Care Setting in Pakistan: A Series of Six Cases

**DOI:** 10.7759/cureus.863

**Published:** 2016-11-04

**Authors:** Avais Raja, Hana Manzoor, Imad-ud-din Saqib, Waqar Jan, Mamoon Rashid

**Affiliations:** 1 Department of Orthopaedic Surgery, Shifa College of Medicine, Islamabad, Pakistan; 2 Department of Neurology, Shifa International Hospital, Islamabad, Pakistan; 3 Department of Plastic Surgery, Shifa International Hospital, Islamabad, Pakistan; 4 Trauma and Orthopaedic Department, Shifa International Hospital, Islamabad, Pakistan

**Keywords:** fvfg, musculoskeletal tumors, tertiary setting, pakistan

## Abstract

**Objective:**

To determine the application, success and complications of the utilization of free vascularized fibular grafts (FVFG) in the reconstruction of lower limb defects after resection of primary lower limb musculoskeletal tumors.

**Methodology:**

This descriptive retrospective case series analysis was conducted at Shifa International Hospital from January 2011 to January 2016. It included patients who had premalignant and malignant conditions of the lower limb and subsequently had the lesion resected followed by FVFG surgery. The data collected was to outline the demographic profile, clinical features, and post-procedure outcomes and complications.

**Results:**

There was a total of six patients. The mean age of the patients was 25.8 ± 11.8 years (range: 15-40 years). The patients presented with pain, swelling, inability to bear weight and/or restriction of movement at the joint. Postoperatively, one patient had proximal wound necrosis and one patient had a thrombus in the anastomosed vessels, both of which were managed successfully.

**Conclusion:**

With a success rate of 100% at the end of the six-month follow-up period, FVFG surgery is a reliable procedure that may be successfully carried out for musculoskeletal tumors of the lower limb with minimal complications.

## Introduction

A free vascularized fibular graft (FVFG) is a graft containing tissue and/or bone of the fibular region with its vascular pedicle that is attached to a recipient site for reconstruction of bone defects [1]. In 1975, Taylor et al. introduced the first FVFG in a limb salvage procedure after a trauma, which revolutionized the procedure worldwide thereafter [2]. Since then, over 600 published articles have been cited on PubMed pertaining to FVFG being the most commonly used free vascularized bone graft [3]. Currently, FVFG has been utilized for the treatment of bony defects resulting from congenital anomalies, infection, tumors, avascular necrosis of the femoral head, and traumatic salvage procedures [3].

Many patients with premalignant and malignant bone tumors undergo wide resection to decrease risk of local recurrence only to leave a large bony defect behind [4-5]. The various methods of reconstruction include massive allograft, distraction osteogenesis, endoprosthetic diaphyseal replacement, and vascularized or nonvascularized bone grafting [4]. Vascularized bone grafts carry the advantage of reconstructing large bony defects due to their independent blood supply that allows integration of the graft into the host with a high union rate [6]. We conducted a case series analysis where a FVFG was used to fill in the intercalary defect in the femur after a wide resection of various premalignant and malignant conditions.

The authors have obtained written informed consent for written and electronic distribution of the report from the patients.

## Materials and methods

This study was carried out as a retrospective case series analysis of lower limb reconstruction by FVFG after resection of the primary musculoskeletal tumor between January 2011 and January 2016 at Shifa International Hospital, Islamabad, Pakistan.

### Subjects selection

Thirty patients were identified who had undergone FVFG surgery in the aforementioned time period. Inclusion criteria included patients who had premalignant or malignant conditions of the lower limb. Exclusion criteria included unavailable radiological investigations, preoperatively or postoperatively, and a follow-up period of less than six months. This left a total of six patients meeting the required criteria.

### Preoperative assessment

A detailed history and examination was carried out at the initial visit to assess the size of the tumor, signs and symptoms the patients presented with, and any evidence of local invasion of adjacent structures or distant metastasis. The patient then underwent x-rays with anteroposterior (AP) and lateral views, computed tomography (CT) or magnetic resonance imaging (MRI) scans of the tumor site, CT scan of the abdomen, and chest x-rays. Thus, the exact size and site of the lesion was identified, and it was ensured that there was no distant metastasis before proceeding with the surgeries.

The cases were presented in a multidisciplinary team meeting involving the orthopedics, plastic surgery, pathology, and oncology departments, and a definite plan was discussed and decided upon, which included surgical resection of the tumor, FVFG reconstruction, and adjuvant or neoadjuvant chemotherapy.

The patients were then prepared for surgery by evaluating them for peripheral vascular disease, deep venous thrombosis, and previous vascular damage along with a referral to an anesthesiologist.

### Surgical technique

In the operation room, the patients were placed in a supine position on a fracture table under general anesthesia. Both legs were prepared and draped appropriately with a tourniquet under the pressure of 300 mm Hg. The respective tumors were excised along with the surrounding structures to ensure tumor-free margins. The specimen was sent for frozen section, and once the margins were found to be clear, the next part of the surgery was carried out.

The FVFG was then taken from the unaffected limb. The fibula was marked, followed by a skin incision anterolaterally parallel to the posterior border of the fibula. Blunt dissection was carried out to the lateral intermuscular septum and fibula between the peroneus and soleus muscles. The fibular vessels were divided. Following that, a distal and proximal osteotomy was conducted of 6 cm from the lateral malleolus and head of the fibula, respectively, and a 6-inch segment of the fibula was harvested with the nutrient vessels and periosteal cuff. The nutrient vessels included the fibular artery and venae comitantes. A double-barrel FVFG was used.

The defect left behind from the excision in the recipient site was thoroughly washed with normal saline and hydrogen peroxide along with curettage from the medullary canal to the cortex. The FVFG was stabilized with an intramedullary nail that was reamed through the proximal and distal bones between the defect, reinforced at both ends with a side plate and screws, avoiding damage to the nutrient vessels of the fibula. Vascular anastomosis of the fibular artery was made with a branch of the femoral artery and the venae comitantes with a branch of the femoral vein using 8-0 Prolene sutures, and secure hemostasis was achieved. Further stabilization was provided using intramedullary nailing, a dynamic compression plate, and screws or K-wires.

The wounds were thoroughly washed with normal saline and closed in layers with 3-0 nylon sutures. The skin was approximated with skin staples. A plaster of Paris (POP) back slab was applied at the end to keep the knee extended.

### Postoperative assessment

The patients were retained in the hospital for six days after surgery, during which they were assessed daily by the plastic surgery department as well as the orthopedics department. A physiotherapist was also involved to assist in early mobilization. The patients were then assessed by an oncologist, and subsequently, five to seven cycles of chemotherapy were given as advised by the oncologist.

The patients were followed up for six months. Along with clinical assessment, radiographs were carried out to look for adequate bone healing, any evidence of prosthetic loosening, any abnormal periosteal reaction, or any lytic or blastic lesions.

## Results

From January 2011 to January 2016, six patients were enrolled in the study. The mean age of the patients was 25.8 ± 11.8 years with a range of 15 to 40 years (Table 1). Fifty percent of the patients were male and 50% were female. None of the patients was diagnosed with any disease earlier or was on any medications.

**Table 1:** Patients included in the case series.

Case	Gender	Age (yrs)	Histopathologic Diagnosis	Site	Size of Tumor (cm)	Site of Graft	Type of Graft	Type of Osteosynthesis	Intervention (in addition to surgery)	Complications
1	Female	40	Giant Cell Tumor	Right Distal Femur (Metaphysis)	7.5x7.5	Left Mid- fibula	Double Barrel FVFG	IntraMedullary Nail	Adjuvant Chemotherapy	Proximal Wound Necrosis
2	Female	17	Fibrous Dysplasia	Left Head and Neck Femur	8.5x5	Right Mid-fibula	Double Barrel FVFG	IntraMedullary Nail	-	-
3	Female	16	Osteosarcoma	Right Shaft of Femur (Diaphysis)	15x2	Left Mid-fibula	Double Barrel FVFG	IntraMedullary Nail	Adjuvant Chemotherapy	-
4	Male	27	Spindle Cell Sarcoma	Right Proximal Fibula and Tibia	21x8	Left Mid-fibula	Double Barrel FVFG	Dynamic Compression Plate and Screws	Adjuvant Chemotherapy	Thrombus in Anastomosed Vessel
5	Male	15	Osteosarcoma	Left Shaft of Femur (Diaphysis)	13.5x9.5	Right Mid-fibula	Double Barrel FVFG	K-wires	Adjuvant Chemotherapy	-
6	Male	40	Osteosarcoma	Left Distal Femur	9x8	Right mid-fibula	Double Barrel FVFG	K-wires	Neoadjuvant Chemotherapy	-

The patients presented with complaints of pain, swelling, inability to bear weight, and/or restriction of movement at the joint. All patients were symptomatic at the time of presentation.

**Figure 1:**
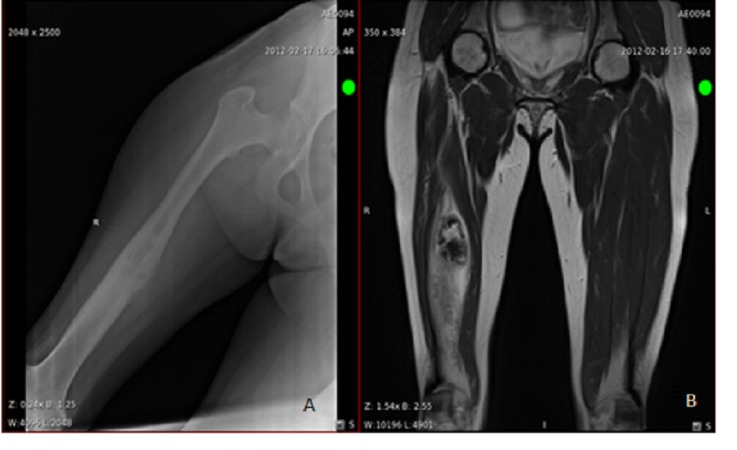
Radiological images of osteosarcoma. Right femur AP view (A), MRI without contrast coronal view (B).

**Figure 2:**
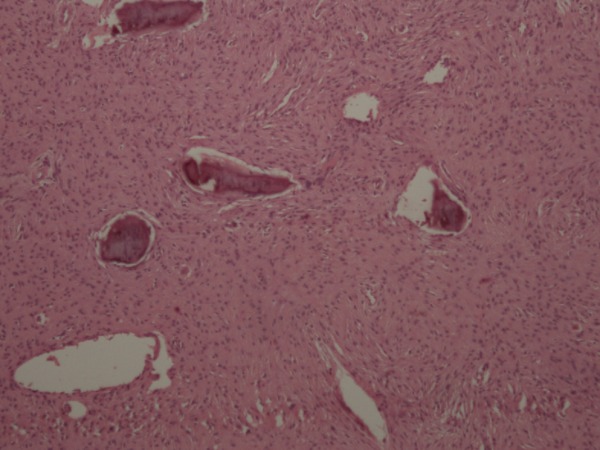
Histological image of osteosarcoma. Nests of tumor cells having lace-like architecture separated from the rest of the tissue. There are also areas of mitosis indicating the aggressive nature of the tumor.

**Figure 3:**
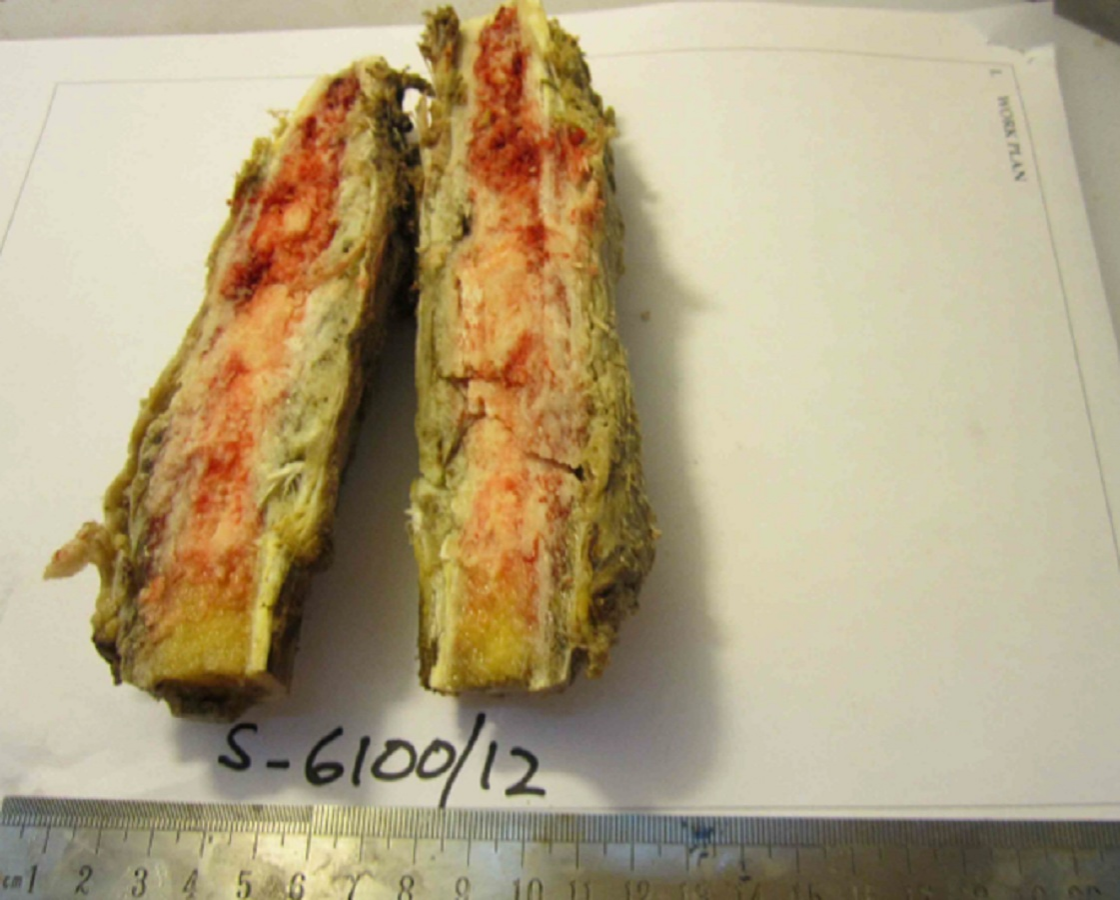
Gross specimen of osteosarcoma on cut section showing intramedullary invasion.

**Figure 4:**
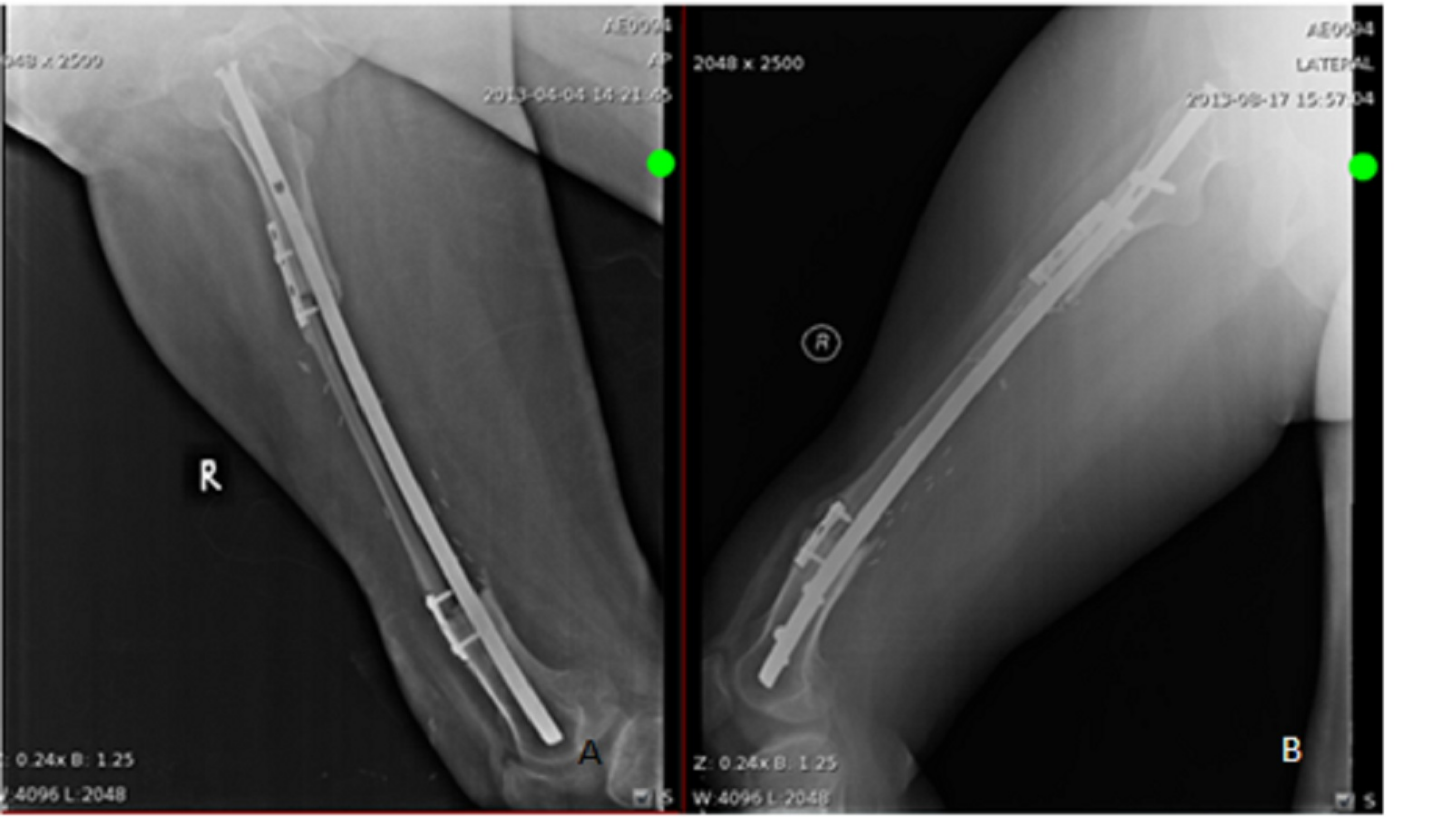
Radiological images showing intramedullary nail and FVFG six-month post-op. AP view (A), lateral view (B).

**Figure 5:**
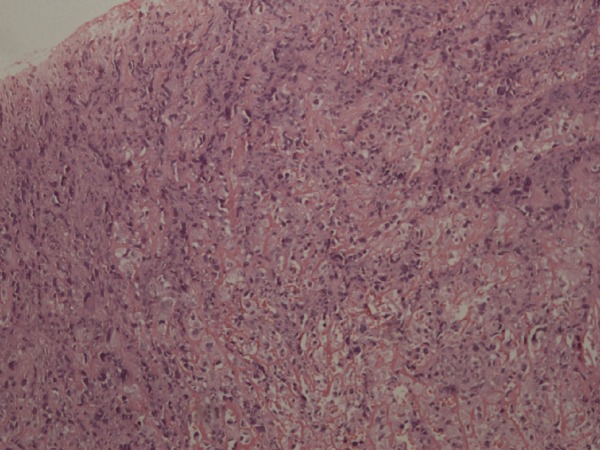
Histological image of fibrous dysplasia. Curvilinear bone trabeculae seen which resemble Chinese characters (irregular, wavy, curved shapes). Note various islands showing increased fibroblastic proliferation.

**Figure 6:**
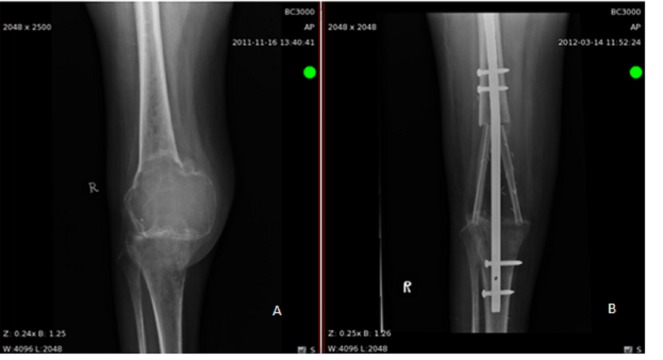
Radiological appearance of giant cell bone tumor of distal femur, AP view (A). Radiological image of intramedullary nail in distal femur and proximal tibia with double-barrel FVFG, three months post-op (B).

**Figure 7:**
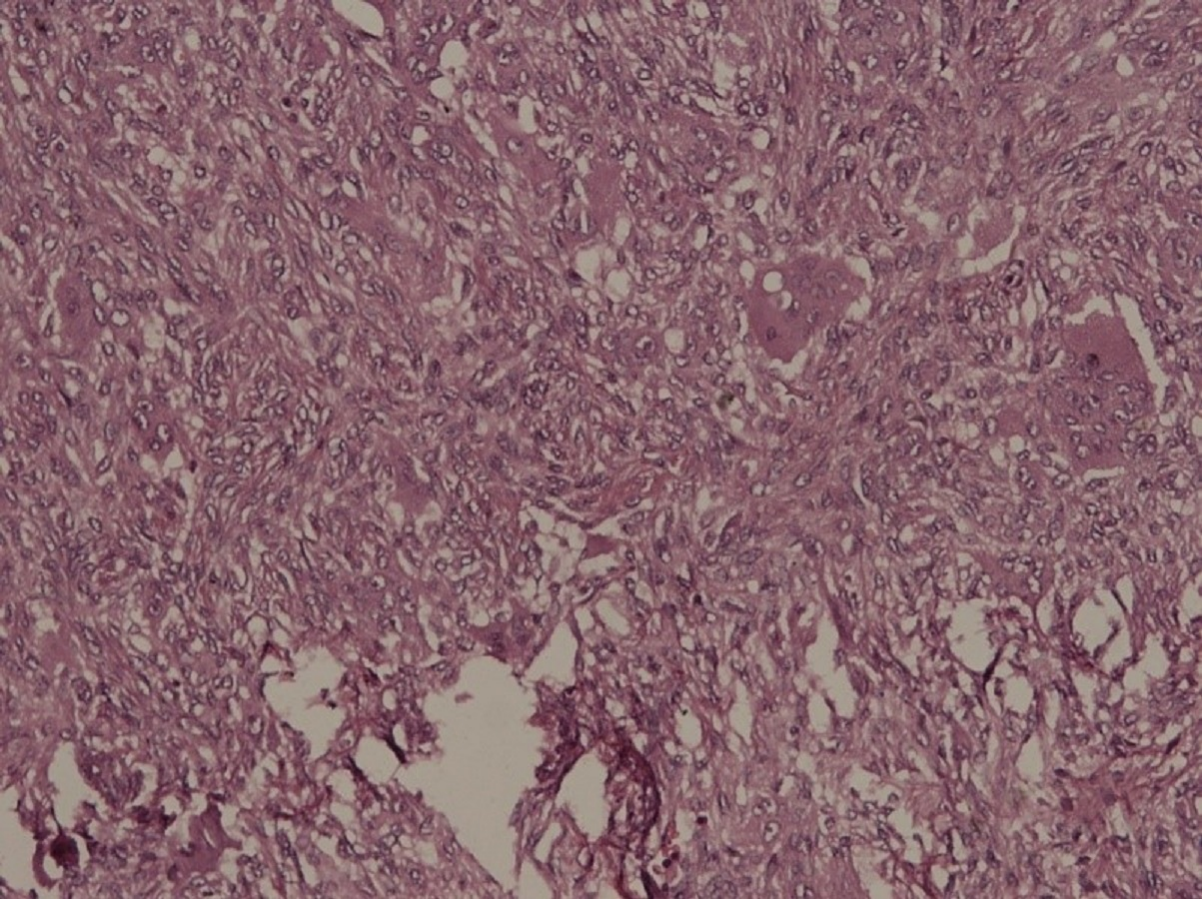
Histological image of giant cell tumor. Abundant giant cells surrounded by nonnucleated stromal cells. Note the abundant giant cells that are light mauve-colored polyhedral structures with basophilic nuclei.

On the basis of clinical judgment and radiological evidence, a clinical diagnosis was made: two patients (33.2%) with giant cell tumor, one patient (16.7%) with osteosarcoma, one patient (16.7%) with Ewing’s sarcoma, one patient (16.7%) with spindle cell carcinoma, and one patient (16.7%) with fibrous dysplasia. Intraoperatively, the clinical diagnoses were revised on the basis of the histopathology reports: three patients (49.9%) with osteosarcoma (Figures 1-4), one patient (16.7%) with spindle cell carcinoma, one patient (16.7%) with fibrous dysplasia (Figure 5), and one patient (16.7%) with giant cell tumor (Figures 6-7).

Following the FVFG series, the complications were divided into intraoperative and postoperative complications. None of the patients had any intraoperative complications. However, postoperatively, one (16.7%) of the patients had proximal wound necrosis, which was debrided and washed thoroughly, and one (16.7%) of the patients had a thrombus in the proximal artery anastomosed, which was discovered on re-exploration on postoperative day one due to unsatisfactory progress of the graft. The thrombus was subsequently removed, and there were no further complications in the six-month follow-up period.

One (16.7%) of the patients was given neoadjuvant chemotherapy while three (50%) of the patients were given adjuvant chemotherapy. The chemotherapeutic agent used was doxorubicin, cisplatin, carboplatin, and methotrexate. One (16.7%) of the patients was given one cycle of doxorubicin, cisplatin, and carboplatin followed by six cycles of methotrexate. Three (50%) of the patients were given five cycles of methotrexate.

## Discussion

What makes the fibula an ideal graft is its gross morphological characteristics that can be modified to reconstruct long bone defects. Being long and straight along with its dual blood supply, the fibula has a vast dimension of fitting into medullary canals of the larger long bones (e.g., humerus, femur, and tibia) to fill in defects up to 26 cm [3, 7]. The composition of the graft may vary to include skin, fascia, muscle, and growth plate depending on the defect [7]. As opposed to a nonvascularized graft, a vascularized graft offers a viable blood supply for osteoblasts to remain alive and to preserve bone remodeling, thus making the graft capable to integrate and hypertrophy [8]. The advantage the FVFG has over other vascularized grafts, such as the iliac crest and the rib, is its durability, strength, versatility, and the ability to undergo various osteotomies [7]. Its applicability in managing diaphyseal defects in the femur using a single- or double-barrel technique with allograft can be efficient. Furthermore, following failed total knee arthroplasty, the FVFG in the knee can be useful in arthrodesis. Other desirable sites for FVFG include defects in the clavicle, humerus, ulna, mandible, tibia, ankle, and cervical and lumbar vertebrae [9].

A long-lasting reconstruction along early fusion of the osteotomies can be induced by the biological properties of the FVFG, leading to low rates of nonunion and fractures, and increasing the rate of internal repair of the allograft [10]. The recommended technique of stabilizing the fibular transplant is the thin-wire fixation method (i.e., Ilizarov method), but nail-plate fixation may be used in the conventional method where intramedullary nail is required [9].

Current literature on the application of FVFG of bone tumors in Pakistan was used in the reconstruction of the mandible, radius, femur, and tibia. Iqbal, et al. in 2014 reported a case of reconstruction of the wrist after excision of a giant cell tumor of the distal radius in a 27-year-old male [11]. Rashid, et al. in 2012 conducted a retrospective study on 18 patients to determine the suitability of mandibular reconstruction of benign pediatric tumors with a FVFG and concluded that it is a good option for reconstruction of benign pediatric tumors [12]. Umer, et al. in 2014 also conducted a retrospective study on nine young adults for the evaluation of reconstruction of upper and lower limb bone tumors with parental allographic FVFG in the pediatric age group ending with the following morbidity: one fracture of the bone due to a nonunion and one big toe drop [13]. Despite this, they considered FVFG to be an acceptable option for reconstruction [13]. Abbas, et al. in 2013 utilized a FVFG for four cases of osteosarcoma of the femur and tibia, out of which one case had a delayed union [14].

## Conclusions

Apart from the proximal wound necrosis and thrombus in the anastomosed vessels, there were no intraoperative or postoperative complications reported. These complications were managed, and in the six-month follow-up period, there was no further morbidity or mortality reported. Thus, FVFG appears to be an excellent option for reconstruction of long bone defects from excision of various musculoskeletal premalignant and malignant conditions.

As far as the private tertiary setting is concerned in Pakistan, FVFG transfer is feasibly performed, but due to the lack of resources in government-based tertiary care hospitals, many patients may not be able to avail the opportunity for an optimal reconstruction.
